# Promoted diffusion mechanism of Fe2.7wt.%Si-Fe10wt.%Si couples under magnetic field by atomic-scale observations

**DOI:** 10.1038/s41598-019-56055-0

**Published:** 2019-12-27

**Authors:** L. J. Fan, Y. B. Zhong, Y. L. Xu, T. X. Zheng, Z. Shen, Z. M. Ren

**Affiliations:** 1 State Key Laboratory of Advanced Special Steel & Shanghai Key Laboratory of Advanced Ferrometallurgy & School of Materials Science and Engineering, Shanghai University, Shanghai, 200072 China; 2 Laboratory for Microstructures, Institute of Materials, Shanghai University, Shanghai, 200072 China

**Keywords:** Magnetic properties and materials, Metals and alloys

## Abstract

Diffusion behavior of newly designed Fe2.7wt.%Si-Fe10wt.%Si couples at 1100 °C for up to 12 h has been investigated under the 0, 0.8 and 3 T magnetic fields. Diffusion thickness of solid solution layer and weight percent of Si on Fe2.7wt.%Si side increase significantly under a magnetic field. Application of a magnetic field promotes the diffusion of solid solution layer through the possible diffusion of vacancies mainly due to the appearance of defects, which has been demonstrated by the increased dislocation density and broadening of the typical XRD peaks. Replacement of Si sits by Fe atoms in the crystal structure leads to the appearance of Fe diffraction peaks, which has been confirmed by the increased interplanar spacings under a magnetic field. The magnetic field benefits the depinning of dislocations and leads to higher dislocation density because of the magnetoplastic effect which has been confirmed by the significantly reduced thickness of Fe2.7wt.%Si. Nano-sized Fe_3_Si particles precipitate in the matrix with an orientation relationship on Fe10wt.%Si side as {220}_Fe3Si_ || {220}_matrix_ & < 1–10 >_Fe3Si_ || < 1–10 >_matrix_. Fe_3_Si particles pin dislocation moving and lead to higher dislocation density.

## Introduction

Silicon steels with silicon content ranging from 2 to 7 wt.% are widely used in power, electrical equipment, telecoms device and so on because of good soft magnetic property^1^. Among the family of silicon steels, the 6.5 wt. % high silicon steel exhibits excellent comprehensive properties such as low magnetocrystalline anisotropy, near zero magnetostriction, low core lose and so on^2–4^. However, the 6.5 wt.% Si steels are difficult to fabricate through conventional rolling process due to the appearance of B_2_(FeSi) and D0_3_(Fe_3_Si) ordered phases which result in embrittlement^5^. In order to manufacture high silicon steels, many methods have been employed, for examples, complex cold or warm rolling process^6^, chemical vapor deposition (CVD) method^7^, improved casting method^8,9^, and so on. Actually, due to the limited production procedures, the silicon content has always been controlled within 4.5 wt.%. For example, the maximum Si content is about 3 wt.% during the cold rolling production of 0.3 mm thick silicon steel. When the Si content further increases to higher level, not only cracking and strip fracture can easily happen, but it will also cause overload of the production equipment. Although the steels can be easily got edge cracking during cold rolling process at room temperature, the CVD method has been applied in limited industrial production^10^. But the SiCl_4_ gas has a strong corrosiveness to equipment and environment at high temperature, and the manufacturing process is also very complex with high energy consumption. As to many other methods, the controllability of production process and manufacturing cost still need to be improved. The electrodeposition combined with heat-treatment method has been proposed by Zhou *et al*., and the new method shows high mass transfer in preparing the near-net-shape steel strips under a magnetic field^11^. All these methods are closely related to diffusion behavior of Fe-Si at high or low temperature, so it is very important to know the specific diffusion behavior and the related mechanisms.

The Fe_3_Si, Fe_5_Si_3_, FeSi, and FeSi_2_ silicides form with the increased Si content at certain temperature from the Fe-Si phase diagram, and the appearance of different phases may have apparent effect on diffusion behavior of Fe-Si diffusion couples. It was confirmed that the stronger affinity existed between neighboring iron and silicon atoms than that between neighboring iron and iron atoms, so it was easier for iron to occupy iron sites than silicon sites^12^. As to the Fe-Si diffusion couples, it is very difficult to distinguish how do the Fe and Si atoms diffuse when many other silicides appear. The diffusivities had been measured for stoichiometric and off-stoichiometric Fe_3_Si at 720 °C and it was found that the diffusivity for Fe_80_Si_20_ was a factor of five to ten times lower than that for stoichiometric Fe_3_Si^13^. The addition of Hf led to the formation of Hf-enriched Hf-Fe-Si precipitate, which was found to play a significant role in effectively suppressing forming of Fe-Si ordered phase^14^. The effect of Fe-17at%P on the alternating current magnetic properties of Fe-3.5 wt%Si alloys was analyzed and it was found that the secondary Fe_3_P phase was detected along the grain boundary, but there was no significant effect of reducing the eddy current loss as an insulation layer^15^. Hence, the types of additive species and forming of the silicides with various structures can lead to dramatically varied diffusivities. What is more, the reaction diffusion in Ni-Al diffusion couples in the steady magnetic field indicates that the magnetic field intensity and direction dependence of growth rate of Ni_2_Al_3_ intermetallic layers can be attributed to the change in number of collision of an atom with neighbors during diffusion, which leads to change the frequency factor for layer growth^16^. Some preliminary results on Fe diffusion in FeSi indicated that diffusion was by orders of magnitude slower than in Fe_3_Si because of the strong influence of structure^17^. So the varied diffusion behavior is also strongly related to the nearby vacancy concentrations. In order to analyze whether the dislocation density in the diffusion couple was changed in a magnetic field, Li *et al*. found that the enhanced diffusivity had been observed in Ni-Al system under the alternating magnetic field due to the increased dislocation density^18^. However, no experimental observation of the dislocations or the evolution of the atomic-scale images with and without magnetic fields had been given.

In previous paper, it was found that the static 0.8 T magnetic field exhibits a suppressing effect on interdiffusion behavior of Fe-Fe50wt.%Si diffusion couples because of the decreased thickness of FeSi and solid solution layer^19^. Different kinds of silicides can easily form when the Si content is higher than 10 wt.%, and now the low Si diffusion couple has been designed to avoid the formation of Fe_3_Si, Fe_2_Si, and FeSi diffusion layers. In order to investigate the diffusion behavior without silicide layers, the effect of a magnetic field on diffusion of low Si diffusion couples has been studied in this paper. The microstructures of both Fe2.7wt.%Si and Fe10wt.%Si sides in the diffusion couples have been analyzed by atomic-scale observations.

## Results and Discussion

**Figure 1:**
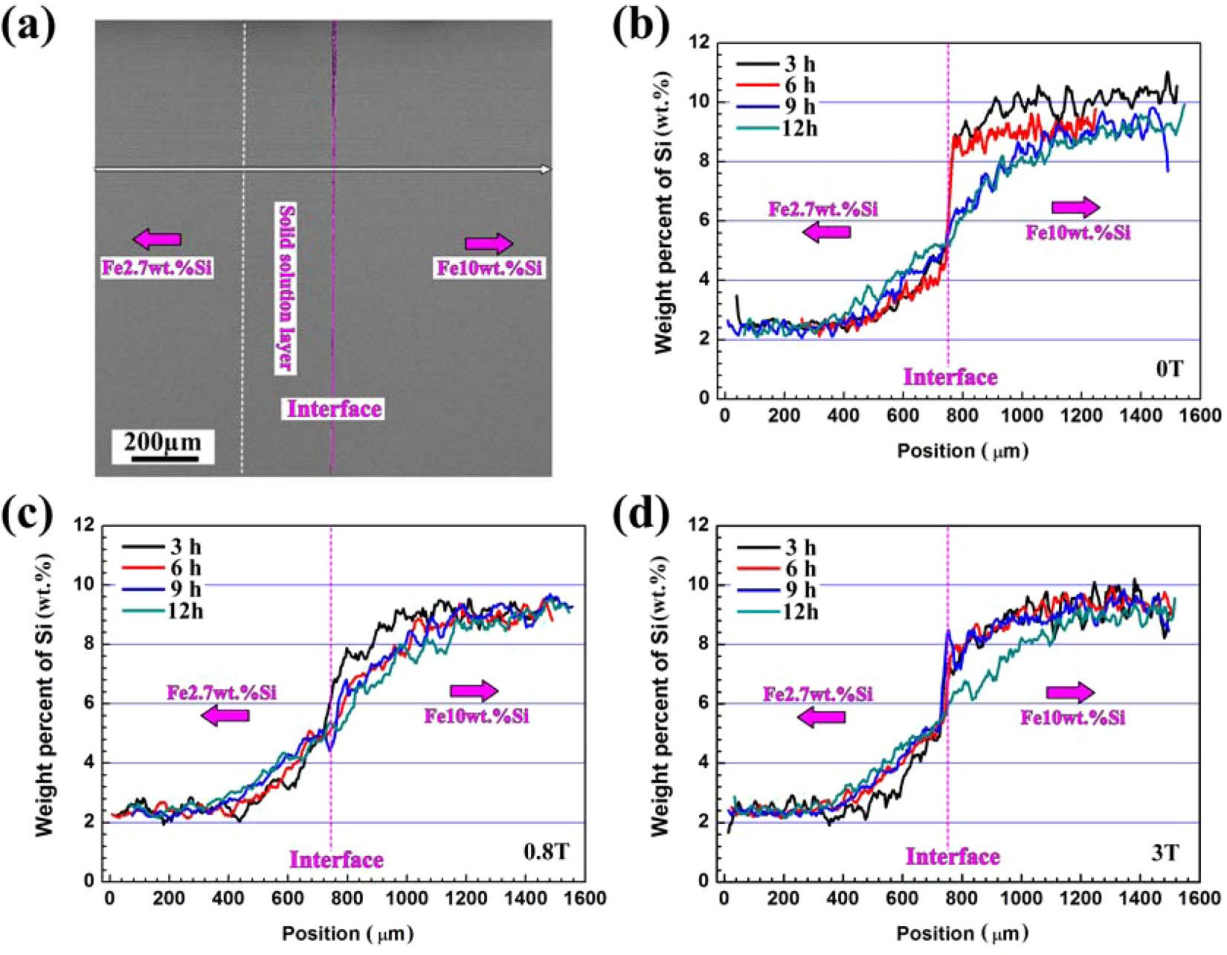
SEM morphology and weight percent of Si in diffusion couples after diffusion at 1100 °C, (**a**) 0.8 T, 3 h, (**b**) 0 T, (**c**) 0.8 T, and (**d**) 3 T magnetic field.

The SEM morphologies and weight percent of Si on both Fe2.7wt.%Si and Fe10wt.%Si sides after diffusion with and without a magnetic field have been analyzed in Fig. 1. The thickness of solid solution layer on Fe2.7wt.%Si side has been measured, and the thickness increases from about 190 μm to 250, 320, and 350 μm after annealing at 1100 °C for up to 12 h without a magnetic field. The values increase significantly with the application of a magnetic field, for example, the thickness of solid solution layer is respectively about 270, 350, 400, and 450 μm after diffusion at 1100 °C for 3, 6, 9, and 12 h under the 3 T magnetic field. The typical cross-section morphology of Fe2.7wt.%Si-Fe10wt.%Si diffusion couple after diffusion for 3 h at 0.8 T is shown in Fig. 1(a). The diffusion “Interface” and two sides of “Fe2.7wt.%Si” and “Fe10wt.%Si” have been marked, and it is very hard to distinguish the solid solution layer and both the matrix of Fe2.7wt.%Si and Fe10wt.%Si according to the SEM image. So the weight percent of Si after diffusion with and without a magnetic field has been analyzed by SEM-EDS. It can be easily to identify the interface after diffusion for 3 and 6 h without a magnetic field, and the concentration gradient of Si has a great difference on both sides of diffusion couple, the weight percent of Si varies very slightly on Fe2.7wt.%Si side, but the value decreases on Fe10wt.%Si side within 6 h (Fig. 1(b)). The concentration gradient of Si has been reduced at the interface after diffusion for more than 6 h, and the Si content increases on Fe2.7wt.%Si side rather than Fe10wt.%Si side with the prolonged diffusion time. After diffusion in the 0.8 and 3 T magnetic fields for 3 h, the diffusion interface is not as obvious as that without a magnetic field. The weight percent of Si increases gradually on Fe2.7wt.%Si side and the concentration gradient of Si reaches similar values, but the weight percent of Si decreases on Fe10wt.%Si side under the 0.8 T magnetic field (Fig. 1(c)). The weight percent of Si near the “Interface” on Fe2.7wt.%Si side after diffusion for 3 h under the 3 T magnetic field is similar with that under the 0.8 T magnetic field, and the weight percent of Si on Fe10wt.%Si side decreases compared with that without a magnetic field (Fig. 1(d)). Although the weight percent of Si increases significantly after diffusion for 6 h on Fe2.7wt.%Si side, the value increases slightly after diffusion for 9 and 12 h under the 3 T magnetic field compared with that under the 0.8 T magnetic field, and the value at the diffusion interface tends stable after diffusion for more than 9 h. After diffusion for 12 h, the weight percent of Si decreases significantly on Fe10wt.%Si side under the 3 T magnetic field. So the application of a magnetic field benefits the diffusion of Fe2.7wt.%Si-Fe10wt.%Si, and the increased magnetic field intensity can reduce the time to obtain a higher concentration of Si on Fe2.7wt.%Si side at certain position of the couples.

**Figure 2:**
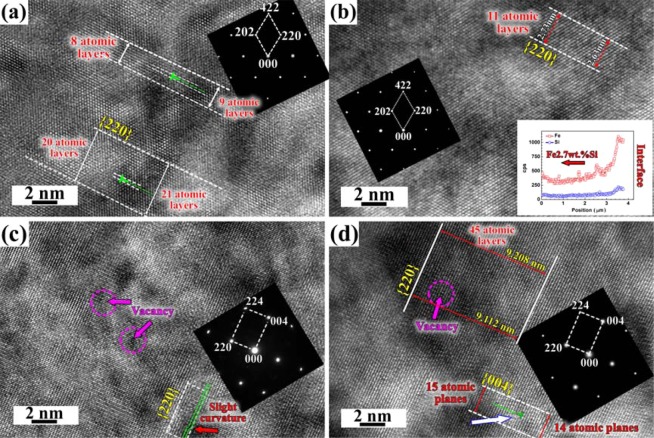
HRTEM atomic-scale images and corresponding SADP after diffusion at 1100 °C for 12 h under the 3 T magnetic field, (**a**) Fe2.7wt.%Si matrix (region 1), (**b**) 5 μm to the interface on Fe2.7wt.%Si side (region 2), (**c**) Fe10wt.%Si matrix (region 3), (**d**) 5 μm to the interface on Fe10wt.%Si side (region 4).

The microstructures have been analyzed and the typical interplanar spacings have also been summarized in Table 1. The HRTEM atomic-scale images on both Fe2.7wt.%Si and Fe10wt.%Si sides after diffusion at 1100 °C for 12 h under the 3 T magnetic field are shown in Fig. 2. It can be clearly seen that Fe and Si atoms in Fe2.7wt.%Si matrix of “Region 1” are positioned orderly (Fig. 2(a)). According to the PDF #06-0696 (Fe), the d{101} and d{211} of pure Fe with body centered cubic crystal structure is about 2.027 Å and 1.170 Å, respectively. So the interplanar spacing of {202} and {422} respectively decreases by about 0.53–0.63% and 0.62% with addition of 2.7 wt. % Si element, which is mainly due to replacement of Fe atoms by Si atoms with smaller atomic radios. Dislocations on {220} atomic planes can be easily observed in Fig. 2(a). The atomic-scale image of “Region 2” on Fe2.7wt.%Si side 5 μm to the interface is shown in Fig. 2(b), and the {220} atomic planes have also been labeled. Compared with those of pure Fe, the interplanar spacings of {202} and {422} increase, and the values are larger than those of Fe2.7wt.%Si matrix (Table 1). Although most of the atomic planes are parallel without significant dislocation near couple interface, the interplanar spacing of {220} varies slightly in some regions, for example, 11 atomic planes distribute between two white dotted lines in Fig. 2(b), the upper left side is about 2.73 nm while the lower right side is about 2.63 nm. The corresponding TEM-EDS also indicates that concentrations of Fe and Si decrease gradually from couple “Interface” to Fe2.7wt.%Si matrix direction. The above results confirm that the evolution of atomic-scale character is strongly related to the distribution of Fe and Si atoms, because weight percent of Si at the interface on Fe2.7wt.%Si side dramatically increases after diffusion (Fig. 1). Fig. 2(c) shows the HRTEM atomic-scale image of Fe10wt.%Si matrix after diffusion at 1100 °C for 12 h under the 3 T magnetic field. Compared with the interplanar spacing of Fe_3_Si phase of d{220} = 2.005 Å, d{004} = 1.418 Å, and d{224} = 1.157 Å according to the PDF #65-0146 (Fe_3_Si), the interplanar spacings of d(220), d(004), and d(224) have been respectively reduced by about 2.38%, 2.71%, and 8.13%. As to the region of 5 μm to the diffusion interface, the weight percent of Si is always lower than that of Fe10wt.%Si matrix, the atomic-scale image after diffusion for 12 h under the 3 T magnetic field is shown in Fig. 2(d). Although the interplanar spacings of (220), (004), and (224) are still lower than those of Fe_3_Si, the values increase compared with those of Fe10wt.%Si matrix. Many vacancies can be easily observed in Fe10wt.%Si matrix, and some {220} atomic planes exhibit a slightly curved distribution (Fig. 2(c)). The edge dislocation line has been marked along {004} atomic planes near diffusion interface on Fe10wt.%Si side, and there are some localized lattice distortions within the region around the dislocation line because of the vacancy (Fig. 2(d)). The existence of vacancy can also lead to the slightly varied thickness of the {220} atomic planes. As a result, defects can easily occur in the diffusion couples under a magnetic field.

**Table 1:** Interplanar spacings of the typical atomic planes in different regions with and without a magnetic field (region 1: Fe2.7wt.%Si matrix, region 2: 5 μm to the interface on Fe2.7wt.%Si side, region 3: Fe10wt.%Si matrix, region 4: 5 μm to the interface on Fe10wt.%Si side).

Magnetic field (T)	Region	d_(202)_ (Å)	d_(220)_ (Å)	d_(422)_ (Å)	d_(004)_ (Å)	d_(224)_ (Å)	d_(2–20)_ (Å)	d_(400)_ (Å)
3	1	2.016	2.014	1.163	/	/	/	/
3	2	2.138	2.119	1.258	/	/	/	/
3	3	/	1.957	/	1.379	1.063	/	/
3	4	/	1.984	/	1.394	1.150	/	/
0	1	1.985	1.938	1.117	/	/	/	/
0	2	2.057	2.025	1.202	/	/	/	/
0	3	/	1.900	/	/	/	1.937	1.332
0	4	/	1.973	/	/	/	1.954	1.356

**Figure 3:**
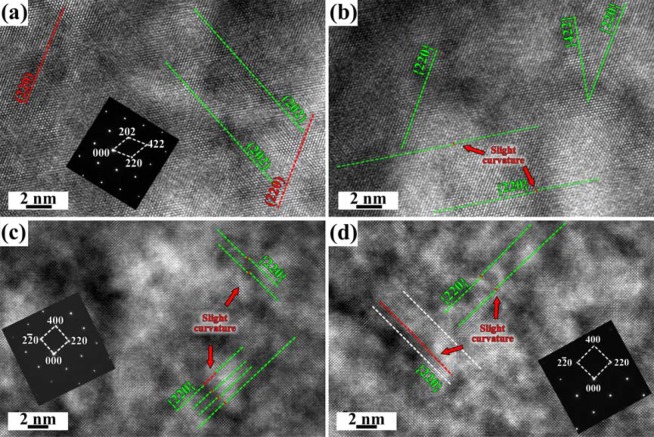
HRTEM atomic-scale images of (**a**) Fe2.7wt.%Si matrix (region 1), (**b**) 5 µm to the diffusion interface on Fe2.7wt.%Si side (region 2), (**c**) Fe10wt.%Si matrix (region 3), (**d**) 5 µm to the diffusion interface on Fe10wt.%Si side (region 4), after diffusion at 1100 °C for 12 h without a magnetic field.

Fig. 3 shows the atomic-scale images on both Fe2.7wt.%Si and Fe10wt.%Si sides after diffusion at 1100 °C for 12 h without a magnetic field. The HRTEM image shows that both the (220) and (202) atomic planes are aligned parallel in Fe2.7wt.%Si matrix (Fig. 3(a)). However, slight curved lattice has been observed along {220} atomic planes near the couple interface on Fe2.7wt.%Si side in addition to the parallel {422} atomic planes (Fig. 3(b)). The crystal zone axis is <001> , the {220} and {400} atomic planes have been identified, and the slightly curved lattice in some regions on {220} atomic planes has also been observed in Fe10wt.%Si matrix and near the interface (Fig. 3(c) and (d)). Compared with the interplanar values after diffusion with and without a magnetic field, all the values under a magnetic field are larger than those without a magnetic field (Table 1). Although the interplanar spacing near interface region is slightly larger than that in matrix, the value on Fe2.7wt.%Si side rather than on Fe10wt.%Si side is slightly larger than those of ideal Fe_3_Si under a magnetic field.

**Figure 4:**
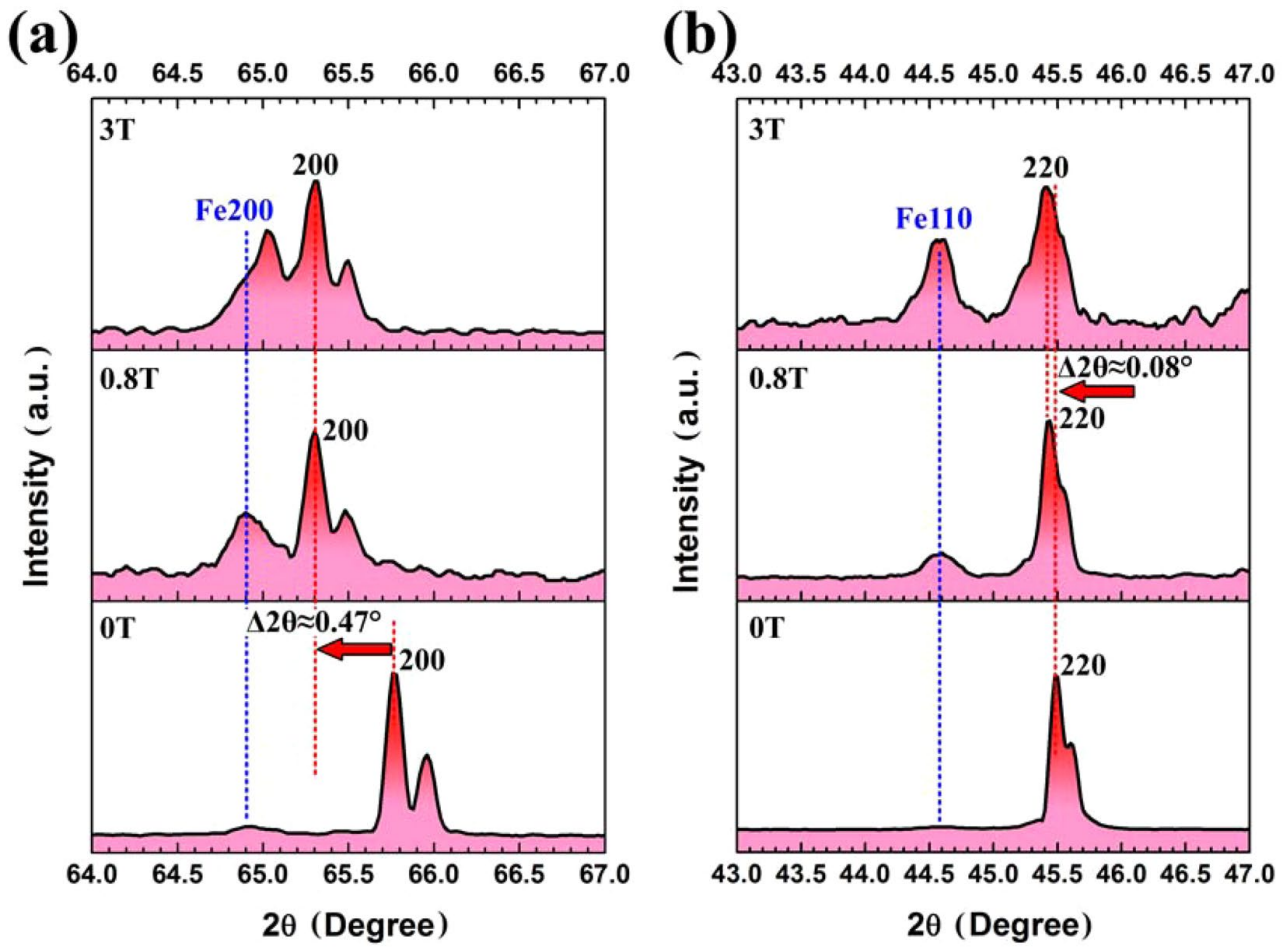
XRD patterns of (**a**) Fe2.7wt.%Si side and (**b**) Fe10wt.%Si side at couple interface after diffusion at 1100 °C for 12 h with 0, 0.8, and 3 T magnetic fields.

The above results indicate that varied concentration of Si at different regions can easily lead to appearance of defects, elastic distortions of the lattice, different interplanar spacing and lattice parameters of the two diffusion couple sides under a magnetic field or not. So X-ray diffraction has been employed to analyze the characters of the typical peaks. The XRD patterns at the interface on both Fe2.7wt.%Si and Fe10wt.%Si sides are shown in Fig. 4. Fig. 4(a) shows that the typical (200) peaks locate at about 2θ = 65.77° without a magnetic field, and the (200) peaks significantly shift to lower 2θ angle side, for example, the 2θ locates at about 65.30° under the 0.8 T magnetic field. While the 2θ angle hardly varies in the 3 T magnetic field compared with that in the 0.8 T magnetic field. Besides, the pure Fe diffraction pattern of {200} can be still observed at 2θ ≈ 64.88° without a magnetic field, and the intensity increases significantly with application of a magnetic field. Fig. 4(b) shows the typical XRD patterns on Fe10wt.%Si side at the interface, the results indicate that the main peaks of {220} vary slightly under different diffusion conditions. The most significant phenomenon is that the Fe{110} peaks with body centered cubic (BCC) structure appear at 2θ ≈ 44.57° with the increased magnetic field intensity. The left shifting of XRD patterns of solid solution layer confirms that the lattice parameters significantly increase under a magnetic field on Fe2.7wt.%Si side, while the lattice parameter of Fe10wt.%Si near the interface hardly varies. It also indicates that the inward diffusion of Fe atoms leads to the replacement of Si by Fe atoms and results in increased interplanar spacing, which can be also confirmed by appearance of the typical BCC Fe peak from XRD patterns under a magnetic field. What is more, the diffraction peak of {200} after diffusion for 12 h both under the 0.8 and 3 T magnetic fields sets nearly the same 2θ angle, it is believed that the position of 2θ angle is strongly related to concentration of Si on Fe2.7wt.%Si side. Because Fig. 1 confirms that weight percent of Si at couple interface under the 0.8 T magnetic field is nearly the same with that after diffusion for 12 h under the 3 T magnetic field, so the {200} diffraction peaks exhibit similar characters under the 0.8 and 3 T magnetic fields.

**Figure 5:**
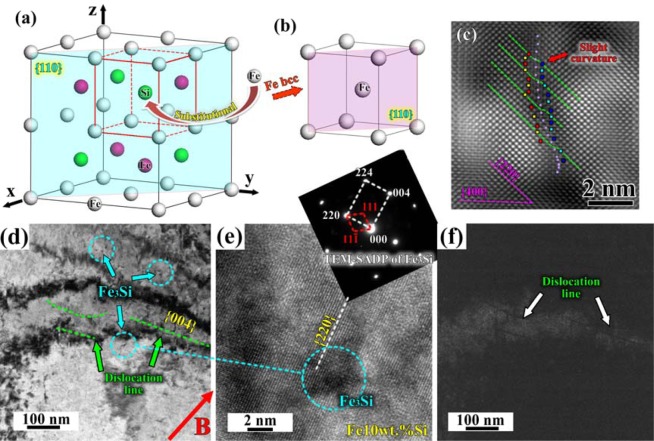
Schematic mechanism of promoted diffusion under a magnetic field, (**a**) unit cell of Fe_3_Si, (**b**) unit cell of Fe with BCC structure, (**c**) inverse fast Fourier transform image of Fe10wt.%Si matrix without a magnetic field, (**d**) bright-field TEM image of nano-sized Fe_3_Si particles and the distribution of dislocation lines, and corresponding (**e**) HRTEM image and SADP of Fe_3_Si, and (**f**) dark-field TEM image of the dislocation lines on Fe10wt.%Si side after diffusion for 12 h under the 3 T magnetic field.

Table 1 shows that the interplanar spacings on Fe2.7wt.%Si side without a magnetic field are smaller than those under the 3 T magnetic field. Fig. 2(a) also shows that the dislocations on {220} atomic planes have been observed on Fe2.7wt.%Si side under the 3 T magnetic field, and the appearance of dislocation contributes to the expansion of unit cell of Fe2.7wt.%Si. This is consistent with left shifting of the {200} XRD patterns by about 0.47° under a magnetic field. The unit cell on Fe10wt.%Si side gets contracted with the addition of 10 wt.% Si element. The lattice parameters can be changed with the different concentration of Si, the replacement of Fe atoms by Si atoms can lead to shirking of the unit cell of crystal structure. But in actually not, because the typical interplanar spacing and lattice parameters on Fe2.7wt.%Si side increase under a magnetic field. As shown in Fig. 1, the average Si content near diffusion interface on Fe10wt.%Si side is slightly higher under a magnetic field, suppose that the crystal structures after diffusion are ideal without any defect, and the atoms in typical Fe_3_Si structure are either Fe or Si, the increased concentration of Si mean the decreased concentration of Fe in the structure. The average diameter of Fe is larger than that of Si atoms, so the lattice parameters of Fe10wt.%Si side at 0 T should be larger than those under a magnetic field. The weight percent of Fe and Si elements at the interface of Fe2.7wt.%Si-Fe10wt.%Si after diffusion at 1100 °C for 3 h with and without a magnetic field has been measured by SEM-EDS analysis, and the results indicate that the concentration of Fe hardly varies. So the promoted diffusion leads to the slightly higher Si content at the diffusion interface, which leads to the increased lattice parameters under a magnetic field. The appearance of BCC Fe diffraction peaks has been discussed in Fig. 5. The Fe_3_Si shows the D0_3_ crystal structure which has a face centered cubic structure, and the unit cell can be thought of 8 BCC subunits with Fe or Si in body centers (Fig. 5(a)). A diffusion mechanism via random nearest neighbor jumping of vacancy can destroy the orderly D0_3_ crystal. The application of a magnetic field can easily induce the appearance of vacancy of Fe or Si or both atoms. When the body centered Si atom was replaced by the nearby Fe atom, the BCC subunit composed of 8 Fe on the cube corners and one in the body center would change into BCC Fe structure (Fig. 5(b)). Both the appearances of Fe{200} and Fe{110} peaks and of the defects indicate the possible vacancy diffusion mechanism of Fe-Si couple. And the increased XRD diffraction peak intensity also indicates that more and more body center positions in the subunits are occupied by Fe atoms. The weight percent of Si near the interface on Fe10wt.%Si side is slightly lower than that without a magnetic field after diffusion for short hours (Fig. 1), which indicates that Si atoms move faster under a magnetic field. The higher Si content on Fe2.7wt.%Si side means more inward diffusion of Si atoms, which indicates that more vacancy will appear under a magnetic field, and the nearby atomic planes will be curved when defects appear, the typical interplanar spacing will also increase. The atomic planes of {220} have been designated as “○” and “□” layers one by one in inverse fast Fourier transform image (Fig. 5(c)), and it can be clearly seen that the varied lattice parameters may lead to the 3–4 atomic layer width curvature. What is more, nano-sized Fe_3_Si particles have been observed on Fe10wt.%Si side after diffusion for 12 h under the 3 T magnetic field (Fig. 5(d)). The HRTEM image shows that the {220} atomic planes of Fe_3_Si distribute parallel with those of Fe10wt.%Si matrix (Fig. 5(e)), and the corresponding TEM selected area diffraction pattern (SADP) confirms that Fe_3_Si particle exhibits an orientation relationship with Fe10wt.%Si matrix on {220} atomic planes. It was found that the precipitated phase was smaller and more dispersive in AA2219 aluminum alloy after annealing under the 0.5 T magnetic field^20^. The precipitate of Fe_3_Si particles with size about 2 nm after diffusion for 12 h is mainly due to the varied chemical compositions, because these particles are not homogeneous in the matrix and Fig. 1(d) also indicates that the weight percent of Si varies significantly in different regions near the diffusion interface. Although the {220} atomic planes around Fe_3_Si particle are parallel in some regions, the nearby atomic planes are slightly distorted along {220}. The corresponding dark-field TEM image shown in Fig. 5(f) indicates that the dislocation lines near the particles are curved along the diffusion direction. So the precipitate of nano-sized Fe_3_Si particles has great influence on the movement of dislocations.

The diffusion behavior of Fe-Fe20wt.%Si couples in the condition without the effects of silicides had been studied, and it was found that the 0.8, 3, and 6 T magnetic fields promoted diffusion of solid solution layer at 1100 °C, and higher magnetic field intensity resulted in thicker solid solution layer^21^. Although weight percent of Si has been decreased to 10 wt. % in this paper, the diffusion thickness of solid solution layer varies slightly under different magnetic fields. For example, the diffusion thickness of solid solution layer under the 3 T magnetic field of Fe2.7wt.%Si-Fe10wt.%Si couple shows similar value with that under the 6 T magnetic field of Fe-Fe20wt.%Si couple at 1100 °C. The concentration of Si on interface and XRD diffraction peaks indicate that the 0.8 and 3 T magnetic fields show slight influence on diffusion behavior of Fe2.7wt.%Si-Fe10wt.%Si. What is more, the diffraction peaks get wider under a magnetic field, and this means that the atomic-scale distributions of Fe and Si gradually deviate from the ideal state with the prolonged annealing time. The broadening and shifting of X-ray diffraction peaks confirm the increased dislocation density, and this leads to the slightly larger lattice parameters of solid solution layer. Wu *et al*. also found that the external magnetic field significantly increased the dislocation density under the 12 T magnetic field^22^. Although the evidences for the interactions among deformation mechanisms of the samples, diffusions behaviors, and the external magnetic fields had been suggested such as the magnetostriction and magnetoplastic effects, the profound understanding of these relationships was far from reached^23^. The magnetostriction would not happen because the annealing temperature was far higher than the Curie temperatures^24^. Magnetoplasticity was observed under the magnetic field of 0.1 to 10 T and was accompanied by several tens or hundreds of percent change in plasticity properties^23^. Li *et al*. found that the enhanced diffusivity under the alternating magnetic field could be ascribed to the increased dislocation density because of the magnetoplastic effect^18^. In the Fe-Fe20wt.%Si diffusion couples, the reduced thickness of pure Fe side had been observed after diffusion at 1050–1150 °C^21^. The thickness of lower Si side of Fe2.7wt.%Si is also thinner than that of Fe10wt.%Si significantly after diffusion at 1100 °C for 12 h. The weight percent of Fe and Si elements at the diffusion couple interface after diffusion for 12 h under the 3 T magnetic field had been measured by SEM-EDS analysis, the results indicated that the weight percent of Fe and Si was respectively about 93.63 wt. % and 6.37 wt.% on Fe2.7wt.%Si side, and that the value was about 92.55 wt. % and 7.45 wt. % on Fe10wt.%Si side, respectively. Compared with the original samples without diffusion, the weight percent of Fe dramatically decreased from about 97.3 wt.% to 93.63 wt.% after diffusion for 12 h under the 3 T magnetic field. There is an about 3.67 wt.% and 2.55 wt.% Fe and Si exchange on Fe2.7wt.%Si and Fe10wt.%Si sides, respectively. So the outward diffusion of Fe atoms leads to the decreased thickness of Fe2.7wt.%Si side, and the inward diffusion of Fe atoms on Fe10wt.%Si side results the occupation of Si sits in the crystal structure by Fe atoms which finally leads to the appearance of Fe{110} patterns. It had been suggested that the magnetoplasticity resulted from “spin micromechanics”, the creation of paramagnetic electron-spin states that affected dislocation motion^23,25^. The effects of electric field intensity on atom diffusion of Cu/Ta/Si stacks during annealing at 650 °C had been investigated and it was found that the enhanced vacancy and dislocation densities under a magnetic field were responsible for the accelerating of atom diffusion^26^. The magnetic field can affect the course of “short” stages of spin-dependent reactions involving defects because only the structural defects may carry a magnetic moment^25^. It was assumed that a magnetic field affected the spin-dependent process of interaction of a dislocation and a paramagnetic point defect at the moment that they moved closer to each other under mechanical stresses, which led to a situation in which the “coupling” behavior of the pairs was replaced by a “decoupling” behavior, thus decreasing the probability of a dislocation being pinned on a obstacles^25^. The magnetic field can depin dislocations from paramagnetic obstacles and thus result in an increase in the dislocation free segment length^27^. This can lead to high dislocation density which has been demonstrated by atomic-scale observations in Fig. 2 and broadening of the typical XRD peaks in Fig. 4. The diffusion rate of solutes along dislocation is always several orders of magnitude than that by lattice diffusion^28^. The promoted diffusion had been observed in Ni-Cr alloys due to the enhancement of the dislocation density, which was also confirmed by the broadening of XRD peaks^29^. Hence, the application of a magnetic field can create more defects include vacancy and dislocation, which can promote Fe and Si atoms movement. Thicker solid solution layer with higher Si content can be obtained after diffusion, and this has great potential for the guidance of manufacturing process design and mass production of high silicon steel.

Diffusion behavior of the newly designed Fe2.7wt.%Si-Fe10wt.%Si diffusion couple at 1100 °C for up to 12 h has been investigated with and without a magnetic field. Application of a magnetic field promotes diffusion of solid solution layer on Fe2.7wt.%Si side through the possible vacancy diffusion, and the increased magnetic field intensity can reduce the time to obtain a higher concentration of Si on Fe2.7wt.%Si side at certain position of the couples. The atomic-scale character is strongly related to the movement of Fe and Si atoms, because Si content at the interface on Fe2.7wt.%Si side dramatically increases after diffusion under a magnetic field. The replacement of Si cites by Fe atoms in the crystal structure leads to the appearance of diffraction peaks of Fe{110}, in which an increased interplanar spacing under a magnetic field is confirmed. Both the limited amount of curved lattice near interface on Fe2.7wt.%Si side without a magnetic field and the curvature for the planes with 3–4 atomic layers thickness on {220} on Fe10wt.%Si side have been observed. However, lots of dislocations with localized lattice distortion appear on Fe2.7wt.%Si side, vacancies and dislocations have also been observed on Fe10wt.%Si side under a magnetic field. Promoted diffusion under a magnetic field is primary due to the appearance of defects, which is proved by an increase in the dislocation density under the influence of a magnetic field and broadening of typical XRD peaks. The magnetic field contributes to the release of dislocations and an increase in their density due to the magnetoplastic effect, which manifests itself in a significantly reduced thickness of the sample Fe2.7wt.%Si. Nano-sized Fe_3_Si particles identified on the Fe10wt.%Si side exhibit orientation relationships with the matrix as {220}_Fe3Si_ || {220}_matrix_ & < 1–10 > _Fe3Si_ || < 1–10 >_matrix_, play the role of pinning of dislocations, and can lead to higher dislocation density.

## Methods

**Figure 6:**
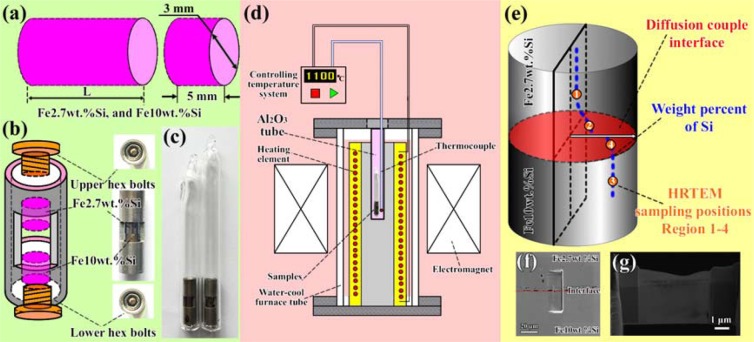
Sample dimensions, sample holders and sampling positions, (**a**) sliced sample preparation after melting, (**b**) interdiffusion couples holders, (**c**) samples sealed by quartz tubes in Ar atmosphere, (**d**) diffusion tests under a magnetic field, (**e**) HRTEM sampling positions of “Region 1” to “Region 4” from the diffusion couples, (**f**) SEM morphology of the diffusion interface after cutting by FIB, and (**g**) the samples for HRTEM observations.

High purity Fe plates and FeSi intermetallic alloys were employed to prepare the Fe2.7wt.%Si and Fe10wt.%Si alloys by the newly designed high frequency induction furnace melting and suction cast equipment. The alloys with diameter of 3 mm were homogeneously heat treated at 1000 °C for 2 h, and electro-cutted into 5 mm thickness by electrical linear machine perpendicular to the length direction (Fig. 6(a)). The samples were polished with no. 400 to 2000 SiC abrasive papers and 1.0 μm diamond paste, cleaned by C_2_H_5_OH, and then dried by hair drier. They were placed in a self-designed stainless steel holder, and the hex cap screw was driven by a tension wrench with the tightening torque about 2 N·m (Fig. 6(b)). The diffusion couples were sealed in quartz tubes in Ar atmosphere (Fig. 6(c)), and respectively annealed at 1100 °C for 3, 6, 9, and 12 h with the 0.8 and 3 T magnetic fields and without a magnetic field (Fig. 6(d)). The thickness and chemical compositions of the intermediate phase layers had been analyzed by scanning electron microscope (SEM) equipped with energy dispersive spectroscopy (EDS). The atomic-scale characterizations were observed by 2100 F high resolution transmission electron microscope (HRTEM). The HRTEM samples preparation positions are shown in the schematic image of Fig. 6(e) which shows that the upside is Fe2.7wt.%Si side. Four regions have been selected to prepare HRTEM samples. The focus ion beam (FIB) using a FEI 600i dual-beam system under conditions of 30 kV was employed to cut HRTEM samples (Fig. 6(f) and (g)). The typical diffraction peaks were analyzed by D/max-2550 X-ray diffractometer (XRD) by using Cu K_α_ radiation.

## Data Availability

The data in this work are available from the corresponding authors on reasonable request.

## References

[1] Garibaldi M, Ashcroft I, Simonelli M, Hague R (2016). Metallurgy of high-silicon steel parts produced using selective laser melting. Acta Mater..

[2] Qin J, Yang P, Mao W-M, Ye F, Liu L (2016). Secondary recrystallization behaviors of grain-oriented 6.5wt% silicon steel sheets produced by rolling and nitriding processes. Acta Metall. Sin..

[3] Pan H, Zhang Z, Xie J (2016). Preparation of high silicon electrical steel sheets with strong {100} recrystallization texture by the texture inheritance of initial columnar grains. Metall. Mater. Trans. A.

[4] Ouyang G, Chen X, Liang Y, Macziewski C, Cui J (2019). Review of Fe-6.5wt%Si high silicon steel-a promising soft magnetic material for sub-kHz application. J. Magn. Magn. Mater..

[5] Xu Y, Zhao G, Wang F, Gao J-W, Xiong T (2016). Effect of heat treatment process on microstructure and texture of hot-rolled sheet of high-silicon steel. Metallogr. Microstruct. Anal..

[6] Wang Y-P (2018). Ultra-thin grain-oriented silicon steel sheet fabricated by a novel way: twin-roll strip casting and two-stage cold rolling. J. Magn. Magn. Mater..

[7] Garibaldi M, Ashcroft I, Lemke JN, Simonelli M, Hague R (2018). Effect of annealing on the microstructure and magnetic properties of soft magnetic Fe-Si produced via laser additive manufacturing. Scripta Mater..

[8] Lu X (2015). Characterization of microstructure and texture in grain-oriented high silicon steel by strip casting. Acta Metall. Sin..

[9] Cui S (2019). Thermodynamic and kinetic analysis of the melt spinning process of Fe-6.5wt.% Si alloy. J. Alloys Comp..

[10] Peng M-H (2018). 6.5wt% Si High silicon steel sheets prepared by composite electrodeposition in magnetic field. J. Mater. Sci. Technol..

[11] Zhou P-W (2013). Effects of parallel magnetic field on electrocodeposition behavior of Fe/nano-Si particles composite electroplating. Appl. Surf. Sci..

[12] Zhang Y, Ivey DG (1998). Fe_3_Si formation in Fe-Si diffusion couples. J. Mater. Sci..

[13] Sepiol B, Vogl G (1993). Atomistic determination of diffusion mechanism on an ordered lattice. Phys. Rev. Lett..

[14] Yu H, Ming K, Wu H, Yu Y, Bi X (2018). Ordering suppression and excellent ductility in soft-magnetic Fe-6.5wt%Si sheet by Hf addition. J. Alloys Comp..

[15] Shin DS, Oh JW, Kim HJ, Park SJ (2018). Microstructural and core loss behaviors of addictive Fe-17at%P based on Fe-3.5wt% Si alloys in powder injection molding. J. Alloys Comp..

[16] Li C-J (2015). Reaction diffusion in Ni-Al diffusion couples in steady magnetic field. J. Alloys Comp..

[17] Mehrer H, Eggersmann M, Gude A, Salamon M, Sepiol B (1997). Diffusion in intermetallic phases of the Fe-Al and Fe-Si system. Mater. Sci. Eng. A.

[18] Li C-J (2017). Enhanced diffusivity in Ni-Al system by alternating magnetic field. Appl. Phys. Lett..

[19] Fan L-J (2015). Effect of static magnetic field on microstructure and interdiffusion behavior of Fe/Fe-Si alloy diffusion couple. J. Alloys Comp..

[20] Liu YZ, Zhan LH, Ma QQ, Ma ZY, Huang MH (2015). Effects of alternating magnetic field aged on microstructure and mechanical properties of AA2219 aluminum alloy. J. Alloys Comp..

[21] Fan L-J (2019). Dual-effects of 6 T strong magnetic field on interdiffusion behavior of Fe-FeSi diffusion couple. Mater Charact..

[22] Wu GH, Hou TP, Wu KM, Chen L (2019). Influence of high magnetic field on carbides and the dislocation density during tempering of high chromium-containing steel. J. Magn. Magn. Mater..

[23] Guillon O (2018). Manipulation of matter by electric and magnetic fields: toward novel synthesis and processing routes of inorganic materials. Mater. Today.

[24] Sajjia, M., Baroutaji, A., Hasanuzzaman, M. & Olabi, A.G. Magnetostrictive cobalt ferrite, nanoparticles preparation and magnetic characterization. *Reference Module in Mater. Sci. Mater. Eng*. (2016).

[25] Morgunov RB (2004). Spin micromechanics in the physics of plasticity. Physics-Uspekhi.

[26] Wang L, Asempah I, Dong S-T, Yin P-P, Jin L (2017). Quantitative studies of electric field intensity on atom diffusion of Cu/Ta/Si stacks during annealing. Appl. Surf. Sci..

[27] Molotskii M, Fleurov V (1997). Spin effects in plasticity. Phys. Rev. Lett..

[28] Mehrer, H. *Diffusion in solid metals and alloys* (Springer, Berlin, 1990).

[29] Li C-J (2017). Alternating-magnetic-field induced enhancement of diffusivity in Ni-Cr alloys. Sci. Rep..

